# Supplementary material for: Human rights violations and associated factors of the Hijras in Bangladesh—A cross-sectional study
Source: PLoS One. 2022 Jul 7;17(7):e0269375. doi: 10.1371/journal.pone.0269375 (PMC9262195; doi:10.1371/journal.pone.0269375)
Supplement: Attachment — Submitted filename: Reviewers comments_26.9.21_sub.docx [file pone.0269375.s003.docx]

**Response to Reviewers comments**

The authors are grateful for the valuable comments that the reviewers provided. Below, we provided a point-by-point response to all comments and indicated in the manuscript using highlighted fonts where changes have been made.

The topic is essential. However, the reviewer has some observations:

1. General: Writing could be improved. Examples are numerous.

**Response: The writing of the revised manuscript has been improved. See the highlighted changes across the manuscript.**

2. The first sentence in the abstract- '..........to preserve the rights of their right' is not clear. It needs to be rephrased.

**Response: Done. The Section was revised.**

3. In the Introduction section, the authors should briefly rationalize why they have measured 'the six human rights violations' among the Hijras in Bangladesh, with appropriate reference(s).

**Response: The following changes have been made;**

The authors selected human rights components by following the significant articles of *The* [*Universal Declaration of Human Rights (UDHR)*](https://www.un.org/sites/un2.un.org/files/udhr.pdf) proclaimed by the United Nations General Assembly in Paris on 10 December 1948 ([General Assembly resolution 217 A](http://www.un.org/en/ga/search/view_doc.asp?symbol=A/RES/217(III))).  Civil, political, economic, cultural, health, and social rights are the legal bindings for the United Nations member states [25].

4. Please split the paragraph under the heading 'Setting'. It is suggested to keep all the texts, from the sentence 'before data collection........', under a different heading 'Data collection'.

**Response: Done. The data collection subheading was created and repositioned in the manuscript.**

5. It is not mentioned how the samples were selected from each district. Equally from each district? (line 153)

**Response: The sentence below was included in the text:**

"To be representative, the equal number of participants were selected from each district".

6. Please briefly write about Bandhu Social Welfare Society (line 154, page 8).

**Response: We have provided some information about the** Bandhu Social Welfare Society**.**

7. Please keep consistency in mentioning the name of the organization 'Bandhu Social Welfare Society' throughout the paper. You have written only 'Bandhu' at page 8 line 156. Better to use acronym (if any).

**Response: We have maintained Bandhu Social Welfare Society across the manuscript.**

8. Please mention the total number of Hijras in the sampling frame provided by the organization. Example, out of XXX, you have randomly selected 346 (line 158).

**Response: The organization provided us with a list of 1600 Hijras. From the list, 346 Hijras were selected randomly.**

9. The paragraph written under the 'Study design' is more about data collection tool/data collection, not about study design. The study is simply a 'cross-sectional' in design. Please move the paragraph under a different heading 'Data collection/data collection tool'.

**Response: The Section has been revised, and the Section on 'study design' section moved into 'data collection.'**

10. It is suggested to write covariates, rather than confounding variables. (line 175).

**Response: Done**

11. Figure 1 is missing! I don't see Figure 1 in the document.

**Response: Figure 1 was included as a separate document in the previous submission. It is now inserted in the main manuscript text for quick visualization.**

12. How is 73% 'about one-third'(line 209). Please make correction.

**Response: This was a typo error and has been corrected**.

13. Different human rights reported under 'Table 2' are not participant characteristics. Please remove the estimates of human rights violations from 'Table 2' and report those under a different Table.

**Response: Table 2 showed the variables used in regression analysis. We have renamed Table 2 to reflect the contents. The new title reads:**

a. Participant Characteristics and the categories of human rights violations (n=346).

**No further change was made in the table**

14. One of the objectives of this study is to identify the factors associated with primary outcomes (violation of human rights). So, this is not clear why the authors have shown the association of one primary outcome with other primary outcomes in univariate and multivariate regression model. Example: Economic right with health rights.

**Response: Agreed and for clarity, now reads "six human rights violation indicators."**
