# Supplementary material for: Human rights violations and associated factors of the Hijras in Bangladesh—A cross-sectional study
Source: PLoS One. 2022 Jul 7;17(7):e0269375. doi: 10.1371/journal.pone.0269375 (PMC9262195; doi:10.1371/journal.pone.0269375)
Supplement: Attachment — Submitted filename: Response_Reviewer2.docx [file pone.0269375.s004.docx]

Response to reviewer’s comments

We thank the reviewer for the very useful comments. We have addressed the comments below and used red fonts to highlight the sections in the manuscript where the requested changes were made.

Reviewer #2:

Question: Methods: Regarding the sample, I believe it needs more detail in the selection process.

Response: we have added the district sampled and indicated the reason for using PPS sampling method in our study.

Question: Needs improvement in the outcome variable. How was it created? This is not clear.

Response: We have added more to this section of the paper for clarity. The revised section now reads:

Outcome variables

The six human rights violations of economic, employment, health, education, political, social/civil rights were the outcome variables in this study. There were three items on the economic right violations, twelve on right to employment, six on right to health, five items each on violations to education and political rights, and eleven on social/civil rights of the participants (see Table 1 for details). For each of the items, a positive response was coded as ‘1’ otherwise ‘0’ for a negative response. A human right violation was recorded if the participant had responded positively to all of the items in that section (i.e. a coding of ‘1’ to all items in that section). For example, on economic right, a violation was recorded only when ‘the items Eco-1, Eco-2 and Eco-2 in Table 1 were each coded as’1’, whereas any coding of ‘0’ in Eco-1, Eco-2 or Eco-2 was considered no violation to the economic right of the respondent. Similar coding method was applied to the employment, health, education, political, social/civil right violations.

Question: Statistical analyses need more detail. How did you carry out the process of creating the models?

Response: We used “collin” command in STATA to check for collinearity and all the variables VIF were less than 4 which indicates that there is no collinearity. We also included all the covariates in the multiple logistic regression analysis at once. We conducted another analytical method by first doing a univariable logistic regression analysis including all the covariates and those with P value < 0.20 were retained and were used to build a multiple logistic regression model. For the multiple logistic regression, a manual backward elimination procedure was applied to remove non-significant variables (P > 0.05). The factors associated with each outcome variables obtained using the two statistical methods were similar with the method reported in the manuscript producing more associated factors. See Table 1 below for when we used the suggested method.

For the reason stated above, no change was made in the revised manuscript.

Table 1 – adjusted odds ratios - multiple logistic regression with retaining those with P value < 0.20

| Variables | Economic rights | Variables | Social and Civil rights |
| --- | --- | --- | --- |
| Demography | AOR[95%CI] | Health right | AOR[95%CI] |
| Dhaka/ Gazipur (Non-Dhaka^^^, OR=1) | 0.11 [0.06, 0.20] | Yes (No, OR=1) | 1.78 [1.10, 2.87] |
| *Hijra Communities* | - | Right to education |  |
| bisexuality (homosexuality, OR=1) | 2.66 [1.29, 5.46] | Yes (No, OR=1) | 2.92 [1.73, 4.92] |
| *Educational status* | - | Variables | Political rights |
| Secondary (Primary, OR=1) | 0.56 [0.26, 1.20] | *District* | AOR[95%CI] |
| Tertiary | 0.26 [0.10, 0.63] | Dhaka/ Gazipur (Non-Dhaka^^^, OR=1) | 0.01 [0.00, 0.01] |
| Living status | - | *Hijra Communities* |  |
| Without family (With family, OR=1) | 2.17 [1.02, 4.58] | bisexuality (homosexuality, OR=1) | 0.02[0.01, 0.06] |
| Variables | Health rights | *Working status* |  |
| Right to education | AOR[95%CI] | Employed (Unemployed, OR=1) | 0.11[0.03, 0.36] |
| Yes (No, OR=1) | 3.76[2.28, 6.19] | Wages in Taka |  |
| Social and Civil right |  | 5000-10000 (< 5000, OR=1) | 1.97 [0.61, 6.31] |
| Yes (No, OR=1) | 1.78[1.09, 2.92] | 10000+ | 5.68 [1.47, 22.01] |
| Political right |  | Health right |  |
| Yes (No, OR=1) | 0.54 [0.34, 0.87] | Yes (No, OR=1) | 0.48 [0.23, 0.99] |
| Variables | Education rights | Employment right |  |
| Employed (Unemployed, OR=1) | AOR[95%CI] | Yes (No, OR=1) | 0.33 [0.17, 0.68] |
| Wages in Taka | 0.28 [0.13, 0.63] | Social and Civil right |  |
| 5000-10000 (< 5000, OR=1) | 1.93 [0.89, 4.20] | Yes (No, OR=1) | 2.58 [1.17, 5.67] |
| 10000+ | 3.11 [1.32, 7.36] | Variables | Employment rights |
| Health right |  | Political right | AOR[95%CI] |
| Yes (No, OR=1) | 3.77 [2.29, 6.22] | Yes (No, OR=1) | 0.57 [0.37, 0.88] |
| Social and Civil right |  |  |  |
| Yes (No, OR=1) | 2.69 [1.57, 4.60] |  |  |

Question: Did you check the final fit of the model? Hosmer-Lemeshow goodness-of-fit method?

Response: Hosmer-Lemeshow goodness-of-fit was not used in this study because it is considered to be obsolete by statisticians for the following reasons: (1) It does not properly take overfitting into account, (2) poor power in small data sets and, (3) dependence on arbitrary binning of predicted probabilities (i.e. the value 10 is arbitrary) and often has power that is too low (Fagerland & Hosmer, 2012; Hosmer, 1997). However and for your record, we have produced the Hosmer-Lemeshow goodness-of-fit for the 6 outcome variables and the result indicated a good fit (P > 0.05)  for all the six human right violations outcomes examined in this study (see Table 2 below). Hence, no change was made to the manuscript base on the limitations discussed above, unless the editor thinks it is necessary as a supplementary table while noting the limitations.

Table 2 Hosmer-Lemeshow goodness-of-fit for the 6 human right outcomes

| Human right variables | chi-square | P-value |
| --- | --- | --- |
| Economic | 7.5 | 0.481 |
| Employment | 11.0 | 0.201 |
| Health | 4.8 | 0.783 |
| Education | 1.6 | 0.980 |
| Social and Civil | 13.4 | 0.100 |
| Political | 10.5 | 0.231 |

References:

Fagerland, M. W., & Hosmer, D. W. (2012). A generalized Hosmer–Lemeshow goodness-of-fit test for multinomial logistic regression models. *The Stata Journal*, *12*(3), 447-453.

Hosmer, D.W. (1997). "A comparison of goodness-of-fit tests for the logistic regression model". *Stat Med*. 16 (9): 965–98

Question: What is the percentage of explanation of the variance of each adjusted model?

Response: We did not report variance for each of our adjusted model because: (1) Logistic regression will always be [heteroscedastic](https://en.wikipedia.org/wiki/Heteroscedastic) – the error variances differ for each value of the predicted score. (2) For each value of the predicted score there would be a different value of the proportionate reduction in error. Therefore, it is inappropriate to think of R² as a proportionate reduction in error in logistic regression (Cohen et al. 2002). However, and for your record, we have produced the R² for the 6 human right outcomes (see Table 3 below). Hence, no change was made to the manuscript base on the limitations discussed above.

Table 3 Maximum Likelihood and Pseudo *R*² for the 6 human right outcomes

| Human right variables | Maximum Likelihood R^2^ | Pseudo R^2^ |
| --- | --- | --- |
| Economic | 0.200 | 0.194 |
| Employment | 0.060 | 0.045 |
| Health | 0.185 | 0.150 |
| Education | 0.191 | 0.157 |
| Social and Civil | 0.115 | 0.095 |
| Political | 0.517 | 0.538 |

Reference:

Cohen, Jacob; Cohen, Patricia; West, Steven G.; [Aiken, Leona S.](https://en.wikipedia.org/wiki/Leona_S._Aiken) (2002). *Applied Multiple Regression/Correlation Analysis for the Behavioral Sciences* (3rd ed.)
