# Supplementary material for: Human rights violations and associated factors of the Hijras in Bangladesh—A cross-sectional study
Source: PLoS One. 2022 Jul 7;17(7):e0269375. doi: 10.1371/journal.pone.0269375 (PMC9262195; doi:10.1371/journal.pone.0269375)
Supplement: Attachment — Submitted filename: Reviewer comments.docx [file pone.0269375.s002.docx]

The topic is important. However, the reviewer has some observations:

General: Writing could be improved. Examples are numerous.

The first sentence in abstract- '..........to preserve the rights of their right' is not clear. Needs to be rephrased.

In the Introduction section, the authors should briefly rationalize why they have measured 'the six human rights violations' among the Hijras in Bangladesh, with appropriate reference(s).

Please split the paragraph under the heading 'Setting'. It is suggested to keep all the texts, from the sentence 'prior to data collection........', under a different heading 'Data collection'.

It is not clearly mentioned how the samples were selected from each district. Equally from each district? (line 153)

Please briefly write about Bandhu Social Welfare Society (line 154, page 8).

Please keep consistency in mentioning the name of the organization 'Bandhu Social Welfare Society' throughout the paper. You have written only 'Bandhu' at page 8 line 156. Better to use acronym (if any).

Please mention the total number of Hijras in the sampling frame provided by the organization. Example, out of XXX, you have randomly selected 346 (line 158).

The paragraph written under the 'Study design' is more about data collection tool/data collection, not about study design. The study is simply a 'cross-sectional' in design. Please move the paragraph under a different heading 'Data collection/data collection tool'.

It is suggested to write covariates, rather than confounding variables. (line 175).

Figure 1 is missing! I don't see Figure 1 in the document.

How is 73% 'about one-third'(line 209). Please make correction.

Different human rights reported under 'Table 2' are not participant characteristics. Please remove the estimates of human rights violations from 'Table 2' and report those under a different Table.

One of the objectives of this study is to identify the factors associated with primary outcomes (violation of human rights). So, this is not clear why the authors have shown the association of one primary outcome with other primary outcomes in univariate and multivariate regression model. Example: Economic right with health rights.
